# Basic concepts and recent advances in nanogels as carriers for medical applications

**DOI:** 10.1080/10717544.2016.1276232

**Published:** 2017-02-09

**Authors:** Iordana Neamtu, Alina Gabriela Rusu, Alina Diaconu, Loredana Elena Nita, Aurica P. Chiriac

**Affiliations:** “Petru Poni” Institute of Macromolecular Chemistry, Iasi, Romania

**Keywords:** Nanogel, response to external stimuli, nanocarrier, drug release, physical and chemical cross linking, genetic material, protein and vaccine delivery

## Abstract

Nanogels in biomedical field are promising and innovative materials as dispersions of hydrogel nanoparticles based on crosslinked polymeric networks that have been called as next generation drug delivery systems due to their relatively high drug encapsulation capacity, uniformity, tunable size, ease of preparation, minimal toxicity, stability in the presence of serum, and stimuli responsiveness. Nanogels show a great potential in chemotherapy, diagnosis, organ targeting and delivery of bioactive substances. The main subjects reviewed in this article concentrates on: (i) Nanogel assimilation in the nanomedicine domain; (ii) Features and advantages of nanogels, the main characteristics, such as: swelling capacity, stimuli sensitivity, the great surface area, functionalization, bioconjugation and encapsulation of bioactive substances, which are taken into account in designing the structures according to the application; some data on the advantages and limitations of the preparation techniques; (iii) Recent progress in nanogels as a carrier of genetic material, protein and vaccine. The majority of the scientific literature presents the multivalency potential of bioconjugated nanogels in various conditions. Today’s research focuses over the overcoming of the restrictions imposed by cost, some medical requirements and technological issues, for nanogels’ commercial scale production and their integration as a new platform in biomedicine.

## Introduction

Taking into account the recent necessities for drastic improvements of current therapies and diagnostic investigations (Etheridge et al., 2013), nanotechnology is implicated in new, more effective pharmaceuticals (Sahoo & Labhasetwar, 2003) as drug delivery systems, imaging techniques, scaffolding and bone replacement, medical tools, cancer, appetite control, diagnostic tests, hormone therapy, cholesterol and immunosuppressant, or different prescriptions to treat particular kinds of illness. In this context, nanohydrogels or the so-called nanogels/hydrogel nanoparticles as multifunctional polymer-based materials with great ability to adapt their properties, gain a great potential in nanomedicine, pharmaceutics and bio-nanotechnology. Due to their unique properties, nanogels are the subject of great interest in multidisciplinary domains, evidenced by the great number of publications on the preparation, properties and applications (Vinogradov 2007; Yallapu et al., 2007; Kabanov & Vinogradov, 2009; Vinogradov, 2010; Ferreira et al., 2013; Singh et al., 2013; Arnfast et al., 2014; Nopphadol et al., 2014; Mavuso et al., 2015; Merino et al., 2015; Molina et al., 2015; Sivaram et al., 2015; Tahara & Akiyoshi, 2015; Wang et al., 2015; Khoee & Asadi, 2016; Soni et al., 2016; Zhang et al., 2016).

Nanogels have a three-dimensional structure formed by chemically or physically crosslinked polymers with hydrophilic or amphiphilic macromolecular chains, able to swell, by holding a great amount of water, with no dissolving but maintaining the structure intact. The great water content correlates with the fluid-like transport properties for the biologically active molecules significantly smaller than the gel pore size.

Nanogels can be composed of a variety of natural polymers, synthetic polymers or a combination thereof, chemically (covalent) crosslinked or physically crosslinked with non-covalent bonds by hydrogen bonds, electrostatic and hydrophobic interactions. The great capacity of absorbing water is attributed to the presence of hydrophilic functional groups, such as –OH, –CONH–, –CONH2– and –SO3H, along the macromolecular chains in the polymer structure.

Usually the authors describe nanogel in terms of crosslinked polymer chains with size up to 100 nm (Sasaki & Akiyoshi, 2010), but the accepted dimensions register up to 200 nm (Bencherif et al., 2009) or more (frequently 1–1000 nm) (Akiyama et al., 2007).

The “NanoGel™” term was introduced for the first time in the papers where it was prepared as (1) a hydrophilic polymer network by chemically crosslinking poly(ethylene glycol) and (2) poly(ethyleneimine) for antisense oligonucleotides delivery (Vinogradov et al., 1999; Lemieux et al., 2000).

Akiyoshi described the first physically cross-linked nanogels using self-assembly of cholesterol-bearing polysaccharides in water through the study of self-organization of amphiphilic polymers; they also applied the physically cross-linked nanogels as nanocarriers for drug delivery systems (Akiyoshi et al., 1993).

Nanogel-based formulations confirm to be a useful scaffold in nanomedicine including: biosensors, artificial muscles, biomaterials, biochemical separation, cell culture systems, biocatalysis, photonics, biomimetics, drug delivery, anticancer therapy, etc. Undoubtedly, cancer is one of the most challenging and studied applications for hybrid nanogels (Yallapu et al., 2011; Dorwal, 2012; Maya et al., 2013; Soni et al., 2016). However, the nanogels were explored from a longer period of time in relation with trends for the synthetic procedures, not only for drug delivery systems but others like quantum dots, MRI contrast agents and other diagnostic agents (Hasegawa et al., 2005; Sun et al., 2005; Gong et al., 2009; Wu et al., 2010; Soni et al., 2016). The variety of polymer systems and the easy designing and tailoring of their physico-chemical characteristics bring the versatile advantage of nanogel formulations. As a delivery system, the nanogels can be used for multiple combinations of drugs for diverse cancers and other immune disorders. The system designed to enclose bioactive substances with different chemical and functional properties, like vaccines, cytokine delivery, nasal vaccines, nucleic acid, creates a new solution in cancer and even autoimmune disease, in future applications. Although more and more scientific papers describing new formulations, synthesis methodologies and potential applications in cancer therapies are appearing, only some variants till now are introduced in clinical trials (Cheng et al., 2007; Kageyama et al., 2008; Aoki et al., 2009; Costantino & Boraschi, 2012). Also, the pre-clinical studies and phase I clinical trials were conducted and the repeated administration in humans (e.g. vaccines) did not prove severe adverse effects, they induced antigen-specific cellular and humoral immunity (Kageyama et al., 2008; Shidhaye et al., 2008; Murphy et al., 2011; Dorwal 2012; Eckmann et al., 2014; Fukuyama et al., 2015; Tahara & Akiyoshi, 2015). However still many safety issues have to be overcome before the results can be applied, although the clinical trials in progress show that they are closer to current practical use.

The review aims at presenting the biomedical nanogels as a new area of research with daily rapid developments, starting from not only the controlled drug delivery, proteins, peptide, and gene delivery, immunological applications, transdermal drug delivery, but also for: quantum dots, MRI contrast agents and other diagnostic agents.

The discussion is focused on some aspects regarding the type of network crosslinking, the main characteristics of nanogel structures that make them suitable for the domain of applications: swelling capacity, stimuli sensitivity, the large surface area, bioconjugation and encapsulation of bioactive substances. Particularly attention is devoted to the most recent work (mostly over the past two years) in the field of nanogels as versatile nanocarriers for: intracellular delivery of genetic material, specific targeted protein delivery and vaccine delivery.

## Nanogels’ succinct overview: rationale for their biomedical use

Nanogels are promising and innovative biomedical systems as dispersions of hydrogel nanoparticles formed from physically or chemically crosslinked polymeric networks that have been called as next generation of drug delivery systems due to their relatively high drug encapsulation capacity, diversity of drugs that can be encapsulated, uniformity, tunable size, ease of preparation, minimal toxicity, stability in the presence of serum, and responsiveness to external stimuli (Oh et al., 2008; Chacko et al., 2012). Naturally-derived nanogels can be prepared from protein polymers, such as collagen, albumin and fibrin, and polysaccharide polymers such as chitosan, hyaluronic acid (HA), heparin, chondroitin sulfate, agarose and alginate (Hoare & Kohane, 2008; Ahmed, 2015).

Poly(lactic acid), poly(lactic)–poly(glycolic) copolymers, polyacrylates and polymethacrylates, poly(e-caprolactone), are some typically synthetic polymers for nanogel preparation. They are mostly spherical particles but the current advancement in synthetic strategies allows for the fabrication of nanogels of different shapes (Rolland et al., 2005; Kersey et al., 2012). They can be also designed to have either a core–shell or a core–shell–corona structure, with at least one of the layers crosslinked for structural integrity.

Being mostly hydrophilic in nature, nanogels are highly biocompatible with a high loading capacity for guest molecules ranging from inorganic nanoparticles to drugs, biomacromolecules like proteins and DNA, with appropriate adaptation of their structure, but without affecting the gel-like performances (Raemdonck et al., 2009; Stuart et al., 2010; Qiao et al., 2011; Chacko et al., 2012). The nanogel has the capacity to encapsulate in the same carrier more than one bioactive substance with different physical properties. This ability is less common to other types of nanoparticles, such as micelles, liposomes, dendrimers or solid lipid nanoparticles (Napier & De Simone, 2007).

Due to the characteristic properties like swelling, stimuli-responsive behavior and softness, the nanogel network protects the encapsulated biological molecules from *in vivo* degradation and elimination, and actively contributes in the delivery process to attain a controlled, triggered response at the target site (Oh et al., 2008; Kabanov & Vinogradov, 2009; Motornov et al., 2010; Zha et al., 2011; Mura et al., 2013; Torchilin, 2014).

**Figure 1.**
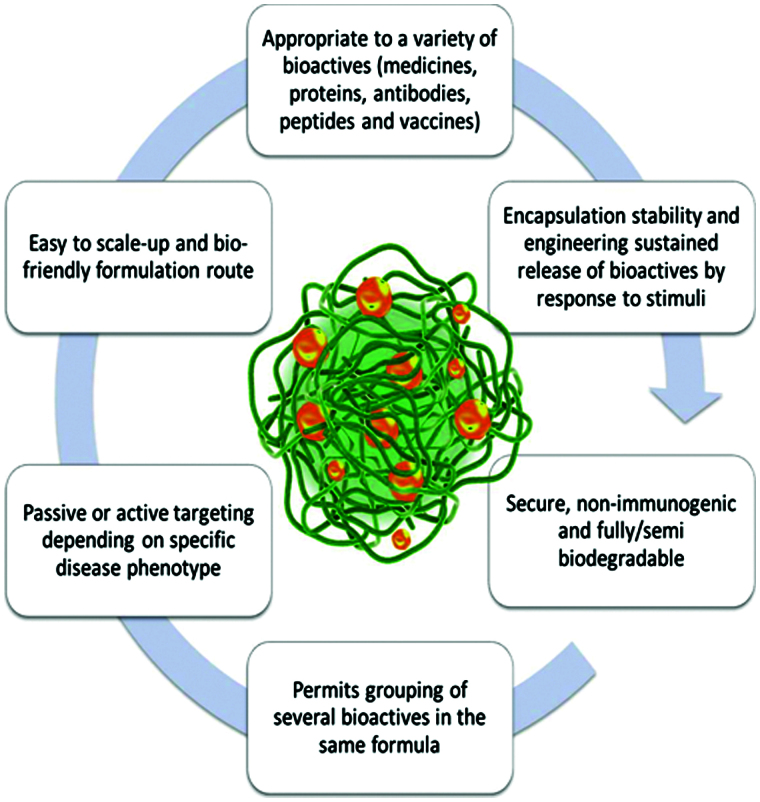
Schematic illustration of potential advantages of nanogel formulations.

Nanogels integrate the features and the potential advantages of hydrogels with those of nanoscale formulations. Some of the representative features of these materials are depicted in Figure 1 (Nomura et al., 2003; Ayame et al., 2008; Hamidi et al., 2008; Kabanov & Vinogradov, 2009; Nochi et al., 2010; Moya-Ortega et al., 2012).

As compared to other bioactive substance delivery systems, nanogels present some unique advantages. They have potential for site targeting and controllable release of bioactive substances, with diminishing side effects. In the range of bioactive substance delivery, similar to hydrogels, high water content/swellability and hydrophilicity, biocompatibility, tunable nanoparticle size, designed physico-chemical-mechanical properties are important characteristics (Ahmed, 2015).

Soni & Yadav (2016) have shown that in balance with nanoparticles such as micelles, biodegradable nanoparticles, liposomes or nanocapsules, nanogels possess a high drug loading capacity and a controlled release modulated by varying crosslinking densities of the polymers (up to 50% of loading efficiency). Furthermore, Vinogradov et al. (2006) developed a drug delivery system for chemotherapy using nucleoside 5′-triphosphate (NTP) as drug model. NTP was encapsulated in a polymeric nanogel composed of branched PEI and polyethylene glycol (PEG)/Pluronic® molecules. NTP is a therapeutic agent, known as the active form of nucleoside analogs (NA) that can inhibit the activity of DNA (RNA) polymerases in proliferating cancer cells. The principal disadvantage when it comes to the systemically administration of NTP is the drug instability and rapid degradation in the blood stream. Initially, other delivery systems such as liposomes were intended to be utilized for this therapeutic agent encapsulation, but various studies indicated a rapid diffusion through the liposome bilayer of similar small hydrophilic molecules like ara-C and 5-fluorouridine. Thus, as it was revealed by Vinogradov et al. (2006), the nanogel network succeeded in increasing the drug stability unlike the liposome and the polymer envelop protected the therapeutic agent from enzymatic degradation.

However, just like the other drug delivery systems, nanogels show limitations regarding the optimization of biodistribution, degradation mechanism and component toxicity. First issue can be overcame by increasing the ratio between the components and thus in this manner, the fast disappearance of nanogels from the bloodstream as a result of RES/macrophage uptake after systemic administration is prevented. Other important features that need to be considered are associated with the biodegradation mechanism of nanogel components that can influence the system cytotoxicity and efficient drug release. Thus, a proper selection of nanogel precursors should be made so as to permit a fast renal exclusion of the degradation products (<40 kDa) (Vinogradov, 2007). Also, to adjust nanogels degradability, labile bonds prone to be cleaved upon exposure to reductive environment inside cells, in the polymer backbone or in the crosslinks of the hydrogel network can be integrated (Tahara & Akiyoshi, 2015).

They are highly stable due to the fact that they are internally crosslinked. They can be highly hydrophilic and in biological environment, they will swell and retain a high level of water/body fluids making them generally biocompatible (Pich et al., 2008; An et al., 2011; Wu & Wang, 2016).

In principle, higher bioactive substance-loading capacities can be expected for nanogels as compared to other nanocarriers, because in their swollen state, a larger inner space is available for the incorporation of drugs or macromolecules (Kabanov & Vinogradov, 2009).

The soft structure of nanogels is another distinctive quality. Hendrickson et al. showed that if a force close to the renal filtration pressure is applied to nanogels, they can pass through pores more than 10 times smaller than their size (Hendrickson & Lyon, 2010). Nanogels softness has a direct influence on their biodistribution and circulation time in the body. Zhang et al. (2012) have prepared nanogels with tunable flexibility based on zwitterionic materials, such as poly(carboxybetaine) by changing their cross-linking densities and solid contents, for constructing long circulating nanoparticles. *In vivo* studies of these nanogels show that softer nanogels because of the deformability pass through physiological barriers, especially the splenic filtration, more easily than the stiffer variants, consequently leading to longer circulation half-life and lower splenic accumulation. Also, the outcomes proved that softer particles can pass through lung tissue better and possess longer circulation time while the stiffer counterparts were mostly entrapped in lung tissue.

At the same time, the smaller tunable size generates conditions for increased blood circulation time after administration, with the possibility of being actively or passively targeted to the desired site of action (e.g. tumor) (Gao et al., 2014), better cellular uptake, reduced uptake by mononuclear phagocytic cells of the reticuloendothelial system (RES) of the organs (Molina et al., 2015).

Also, the large surface area offers a proper space for functionalization and bioconjugation, while the interior network is suitable for the entrapment (encapsulation) of bioactive substances (Kabanov & Vinogradov, 2009; Asadian-Birjand et al., 2012).

In addition, the simplicity of formulation in the majority of cases, diversity of preparation techniques currently available and emerging as advanced technology, the stability of the resulting dispersion are data that cannot be overlooked.

The swelling in an aqueous environment of a nanogel is the most important property. It is controlled by structural features, such as: chemical structure of the polymer matrix, crosslinking degree, charge density in the polyelectrolyte gels, and environmental variables as external triggers. Nanogels undergo an inherent and periodic coil-globular oscillation or swelling and deswelling oscillation in response to external stimuli. They swell because of the solvent penetration into free spaces and undergo rapid volume changes.

By the optimization of their molecular composition, nano-scaled dimension and morphology, the nanogels can be tailor-made to sense and respond to environmental changes in order to ensure spatial and stimuli-controlled release of bioactive compounds (Hoffman et al., 2002; Oh et al., 2007; Yallapu et al., 2007; Liechty et al., 2010; Li & Guan, 2011; Liha et al., 2015; Karimi et al., 2016). Also, by using the stimuli-responsive polymers capable of responding by a phase transition to diverse medically relevant stimuli changing, nanogels will be able to have an intelligent behavior (Börner et al., 2010; Cuggino et al., 2011; Jochum & Theato, 2013; Steinhilber et al., 2013; Giulbudagian et al., 2014; Molina et al., 2014; Tong et al., 2014). This property brought a considerable impact in the use of nanogels like biomedical delivery systems, comparative to the conventional ones (Whitcombe et al., 1997; Ganta et al., 2008; Maharjan et al., 2008; Yarin, 2008; Musyanovych & Landfester, 2014).

As a response to external variables such as changes in temperature, ionic strength, pH, electric and magnetic fields, light, solvent composition, concentration of a specific molecule or bioactive compound (glucose, enzyme) (Hamidi et al., 2008; Ganesh et al., 2014; Crucho, 2015; Tian et al., 2016; Wang et al., 2016; Zhou et al., 2016), the nanogels can react in several ways by altering shape and dimension, solubility, wettability, color, conductivity, light transmitting abilities and surface characteristics.

The degree of response of such polymers can be trigged and controlled by the intensity of the applied stimuli. Typically, the changes are limited to the formation or removal of secondary forces, such as hydrogen bonding, hydrophobic effects, osmotic pressure, electrostatic interactions, etc. (Koetting et al., 2015). Nanogels show much faster responsiveness as compared to the conventional hydrogels because of the small dimensions (Soni et al., 2016).

The most important nanogel systems from the biomedical point of view are those sensitive to temperature and/or pH. The swelling and collapse capacity of the nanogels is distinctive and provides multiple benefits for designing optimal drug loading and release of drugs. Thus, the nanogel networks allow the stimuli-controlled release of encapsulated biologically active compounds including drugs and other biopolymers. Furthermore, nanogels can be chemically modified to incorporate various ligands for targeted drug delivery or triggered drug release (Asadi & Khoee, 2016).

By internal crosslinking modulation of the nanogel network, the drug release can be controlled in response to a stimulus, such as the change in the physiological fluids under disease circumstances (Yallapu et al., 2007). Drugs encapsulated through non-covalent links can be released from the delivery vehicle that reacts to stimuli by a physical change in structure.

**Figure 2.**
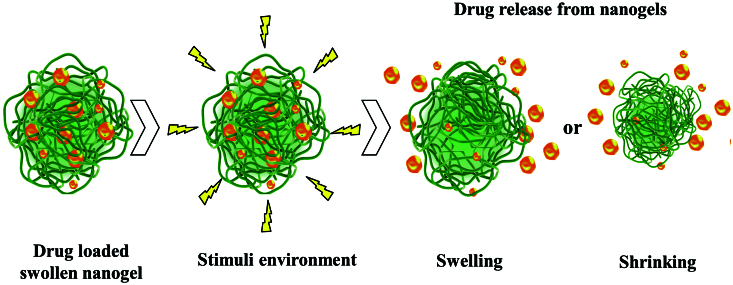
The schematic of drug release from the nanogel network (adapted from Yallapu et al., 2007).

In Figure 2, the process of swelling/shrinking of the nanogel network under the environmental stimuli action with the controlled release of bioactive substances, is represented.

Drug release from nanogel depends on the interaction of hydrophobic, hydrogen links, complexation and/or coordination of drug molecules with the polymer chain networks. The nanogel network characteristics such as the cross-link density of the gel network, the molecular weight of the polymer, the gel network degradation rate and the interaction of the drug–biomacromolecule with the polymer chains in the gel network control the drug release profile (Hoffman, 2002; Vinogradov et al., 2002; Yallapu et al., 2007; Yallapu et al., 2011; Jaiswal et al., 2014).

The three-dimensional cross-linked structures in thermoresponsive polymeric nanogels allow water molecules to interact with hydrophilic groups of the polymer segment helping it to swell while keeping the original structure. This property is manifested near the lower critical solution temperature (LCST) when it turns hydrophobic and removes the water content. As a consequence, the size of the nanogel particles decreases and the therapeutic agent is released via temperature triggered stimulus (Satarkar & Hilt, 2008; Demirel et al., 2009).

For clinical applications, one of the most studied polymer based nanogel with temperature sensitivity is poly(*N*-isopropylacrylamide) because of its biocompatibility. In the LCST domain (32–34 °C), this structure displays coil to globule conformational changes and vice-versa associated with reversible formation and breakage of the hydrogen bond interaction between the polymer and water molecules (Satarkar & Hilt, 2008; Yallapu et al., 2011).

The nanogels containing pH-dependent hydrophobic/hydrophilic repeating units or networks are considered to be better drug delivery carriers. These nanogels having ionizable repeating functional groups can absorb positively charged drugs at alkaline pH through electrostatic attractions and can release them in acidic pH. In the same way, nanogels can absorb biomolecules with low polarity in acidic pH when the core of nanogels is relatively hydrophobic and can release them in neutral pH where nanogels become more hydrophilic. They change their swelling or particles size with respect to pH (Das et al., 2006; Dupin et al., 2006; Pich et al., 2006; Pujana et al., 2014; Zeng et al., 2016).

Tan et al. (2008) synthesized crosslinked structures based on methacrylic acid-ethyl acrylate polymers insoluble at low pH value that present polymeric chain repulsions by increasing pH, because of the acidic groups ionization. This is favorable for particular release of procaine hydrochloride.

Other case is offered by pH sensitive polyacrylic acid chains (Wu et al., 2010) that swell and show a controllable release of temozolodine anticancer drug. Oh et al. (2010) carried out the grafting of diethylaminopropyl groups to glycol chitosan nanoparticles to induce the pH sensitivity with the aim of releasing doxorubicin from the crosslinked nanogel.

Jaiswal et al. (2014) showed in their study that for biomedical applications wherein the therapeutic agents are delivered under specific physiological conditions, always more than one stimulus sensitive nanogel system is required. They synthesized a dual stimuli pH and temperature-responsive nanogel based on poly(N-isopropylacrylamide)-chitosan for carrying and delivering an anti-cancer drug – doxorubicin at specific sites, triggered by one of the stimuli. Chitosan a natural biopolymer with pendant amino groups may be an option because it protonates and solubilizes in weak acidic medium. Supplementary, by grafting of chitosan to poly(N-isopropylacrylamide) segments, a shifting to higher temperature of LCST is obtained. Thus, a nanogel based on poly(N-isopropylacrylamide)-chitosan can form a dual pH and temperature stimuli-sensitive network.

The nanogel networks based on poly(acrylic/methacrylic) and poly(N-isopropylacrylamide) macromolecular chains showed a faster increase in the hydrophilicity and LCST at all the pH values, mainly at pH lower than 5 (Das et al., 2006).

Polyelectrolyte nanogels simultaneously carrying anionic and cationic groups along the polymer main chain, with no particular mutual correlation between them, are called polyampholyte nanogels. Their properties are highly interesting due to the presence of oppositely charged groups in the polymer network and the behavior of such macromolecules is much more pH-sensitive than conventional polyelectrolytes (Das et al., 2006; Yallapu et al., 2011).

In polyampholyte nanogels, anionic and cationic sites may be scattered randomly along the polymer chains, one charged species may outnumber the other one, or one of the charged species may be present only in a narrow pH-range. Therefore, polyampholytes typically bear an overall net positive or negative charge that may vary with pH and ionic strength of the system (Kihara et al., 1998; Laschewsky, 2014; Ekkelenkamp et al., 2016).

## Nanogel preparation techniques

Nanogel networks based on synthetic or natural polymers can be mainly classified into two categories according to their crosslinked structure: chemically crosslinked nanogels which form crosslinking by covalent bonds and physically crosslinked nanogels which form self-assembling through weaker linkages by non-covalent bonds. Crosslinking due to chemical interactions leads to permanent, stable and rigid link in the polymer network. Physical interactions are obtained by polymer chain entanglements or by physical interactions, such as: hydrogen bonds, electrostatic, van der Waals and hydrophobic interactions (Jen et al., 1996; Amamoto et al., 2011; Sood et al., 2016). While the chemical nanogels are difficult to change, in the physical nanogels the sol–gel transitions can precede as a result of the environment stimuli changes.

Due to the multitude of potential applications, a lot of research in designing and synthesizing of the nanogels is in progress. As a result, in the last decade comprehensive presentations of the nanogel methods of preparation are reviewed (Vauthier & Bouchemal, 2009; Chacko et al., 2012; Zhang et al., 2015; Khoee & Asadi, 2016).

In this context, polymer synthesis domain offers the options of different techniques in getting products that meet the relevant medical parameters: size, shape, yield. These methods have their specific positive aspects but limitations, too. The review makes only a short presentation of them.

In the synthesis of nanogels with narrow size distribution of the particles, the stability of the gel particles in dispersion is a very important feature in relation with biomedical applications (Motornov et al., 2010; Smith & Lyon, 2012).

This stability is influenced by the control of the particles’ size, nature and chemical composition of the polymer matrix and the crosslinking type of the polymer chains. While the chemically crosslinked nanogels are attractive because of the reproducibility and size stability, in the physical crosslinking by non-covalent interactions between polymer chains, the weak field strength affects the stability of gels and the control over size during synthesis (Sasaki & Akiyoshi, 2010).

The nanometer-scale in nanogels can be created according to two major approaches: “top-down” and “bottom-up” (Moya-Ortega et al., 2012).

The “top-down” approach generates nanoparticles from large particles or clusters by physical, chemical or mechanical methods such as imprint photolithographic techniques (Particle Replication in Nonwetting Templates, PRINT) (Rolland et al., 2005; Oh et al., 2008). The undesirable problem with “top down” approach is the imperfection of particles’ surface. Also, the method having now few references is more appropriate for synthesizing micron-sized particles (Nie et al., 2005; Kim et al., 2007).

**Figure 3.**
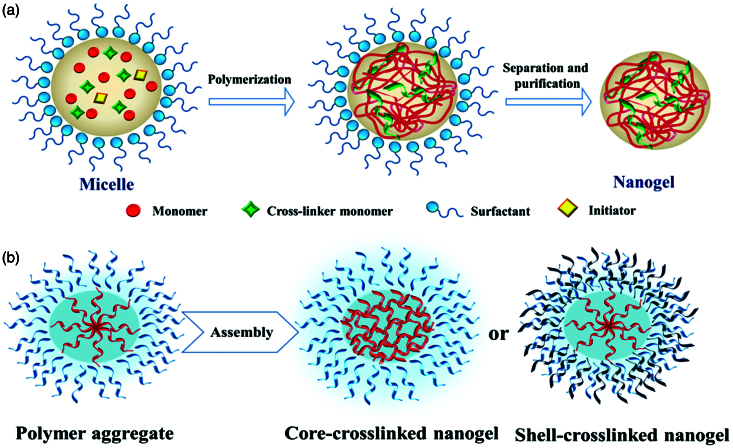
Schematic illustration of the nanogel network created by: (a) direct polymerization of monomers; (b) assembling of a polymer precursor (adapted from Chacko et al., 2012).

The “bottom-up” approach is realized by designing molecular structures and assemblies, starting from molecules or clusters that are subsequently cross-linked by chemical or physical bonds. Practically, the most convenient and common way is achieved *via* classically direct cross-linking copolymerization of monomers or from polymer precursors by assembling them, as it is illustrated in Figure 3.

### Nanogel preparation by free radical crosslinking polymerization

Classically uncontrolled free radical crosslinking copolymerization of monomers combines the two processes of copolymerization and crosslinking in one reaction. In this case, a bifunctional crosslinking agent such as methylene-bis-acrylamide, divinyl benzene, etc. is used to create the crosslinking. The important aspect in this process is to avoid the macrogelation by using “templating” methods based on heterogeneous polymerization in colloidal environments, such as: miniemulsion and microemulsion, or dispersion and precipitation polymerization (Oh et al., 2008,2009; Chacko et al., 2012).

Most of the reported polymer syntheses are performed via free-radical polymerization. These reactions occur only in the nanodroplets formed by the surfactant and the co-surfactant capable of producing monomer micelles as minireactors for the subsequent reaction. The reaction parameters (amount and type of the surfactant, power and duration of the shear stress) determine the nanodroplet dimensions, which will finally assure the nanogel particles’ size, size distribution and the stability (Tobita & Yamamoto, 1994; Grohn & Antonietti, 2000; Tobita et al., 2000; Landfester, 2006; Kabanov & Vinogradov, 2009; Asadian-Birjand et al., 2012).

These methods differ in the selection of particular conditions, but the main principle is the same as emulsion polymerization. Table 1 groups together the uncontrolled free-radical polymerization techniques and highlights some advantages and limitations of every category.

**Table 1.** Uncontrolled free-radical polymerization techniques for nanogel synthesis.

Reaction	Details	Advantages//limitations	References
Miniemulsion	Nanodroplets formation through high shear stress (ultrasonication) of the mixture of monomers and surfactants	Narrow size distributions for diameters in the 50–500 nm range.Allows *in situ* encapsulation/**/**Surfactant and co-stabilizer required.Special equipment necessary (ultrasonic device).	^a^
Microemulsion	Absence of high shear stressUse of a critical concentration of surfactantMonomer molecules are in micelles	Usually nanogel sizes between 10 and 150 nm can be achievedNo shear stress necessary/**/**High surfactant concentration needed.Co-surfactant necessary.	^b^
Dispersion	Initially all the reaction ingredients are soluble in the reaction mediumPolymerization occurs in a homogeneous phaseThe polymers are insoluble and form a stable dispersion with an aid of colloidal stabilizers	Simple batch synthesis.Particle size adjusted by monomer and dispersant concentration in the range of 0.1–15 mmPreferably for core–shell particles synthesis/**/**Preferably for vinylic functionalized monomers	^c^
Precipitation	Initiation of reaction occurs in homogeneous solution of the monomers in the reaction medium.Polymer is soluble in the reaction medium.Particles separation by crosslinking	Batch synthesisNo surfactant requiredParticle size adjusted by monomer concentration in the range of 100–600 nm/**/**Frequently irregular shape and high polydispersity	^d^

^a^(Landfester, 2003; Karasulu, 2008; Crespy & Landfester, 2010; Landfester et al., 2010; Weiss & Landfester, 2010; Landfester & Musyanovych, 2011; Asadian-Birjand et al., 2012; Khoee & Asadi, 2016).

^b^ (Landfester & Musyanovych, 2011; Asadian-Birjand et al., 2012; Khoee & Asadi, 2016).

^c^ (Asadian-Birjand et al., 2012; Khoee & Asadi, 2016).

^d^ (Asadian-Birjand et al., 2012; Khoee & Asadi, 2016).

Heterogeneous controlled/living radical polymerization techniques were also explored in the synthesis of nanogels, including atom transfer radical polymerization (ATRP) and reversible addition fragmentation chain transfer (RAFT). Other researches used ATRP in an inverse microemulsion for the synthesis of stable cross-linked nanogels of water-solublepolymers (Oh et al., 2006,2007). By using ATRP in inverse miniemulsion, nanogels with superior properties in terms of water affinity, degradation behavior and colloidal stability were prepared compared to those obtained by free radical polymerization (Siegwart et al., 2012).

Reversible addition fragmentation transfer process in a single step for the preparation of PEGylated poly (N,N′-dimethylaminomethyl methacrylate) nanogel in the presence of an amphiphilic macro RAFT agent, was used by Dorwal (2012) and Yan & Tao (2010).

### Nanogel preparation from polymeric precursors

Amphiphilic copolymers in solution have the property of self-assembling through the functional groups along the macromolecular chains. By locking this assembly, they can obtain covalently crosslinked networks through crosslinking reaction of the polymeric precursors derivatized with polymerizable groups. Thus, a pioneer paper of Edman et al. (1980) reported the formation of hydrogels from polymerizable dextrans, by reacting it with glycidyl acrylate and polymerizing a solution of the resulting compound.

Starting from polymer precursors, assembled and crosslinked to obtain nanogels, it is possible to tune the particle size by varying polymer concentration and utilizing the LCST behavior of polymers (Chacko et al., 2012).

Some functional groups (disulfide, amine and imine), presence or click chemistry and photo-induced crosslinking provide methods to synthesize nanogels starting from polymer precursor.

In Table 2 are summarized the strategies of nanogels obtaining by starting from polymeric precursors, by evidencing some details of the reactions and the particle size of the synthesized nanogel.

**Table 2.** Main techniques for nanogel obtainment from polymeric precursors.

Reaction	Details	Particle size (nm)	References
Disulfide crosslinking	– Reacting groups: thiol and disulfide, at pH > 8, mild reaction conditions, ease of further functionalization– Self-crosslinking amphiphilic random copolymers (PEG hydrophilic unit and pyridyl disulfide hydro-phobic and crosslinkable unit)	40–60	Zhang et al. (2015)
Amide crosslinking	– Reacting groups: amino and carboxylic, esters, iodides– No additive needed– Adjustable crosslinking degree	50	Zhang et al. (2015)
Imine crosslinking	– Schiff-base reaction– Aldehyde and amine or hydrazide– No catalyst– Mild reaction conditions	6.3–50 function of Mw of PEG	Tan et al. (2012)
Copper-free click chemistry crosslinking	– Reacting groups: alkyl units with amino groups immobilized to the particle shell *via* amidation of hydrophilic polymer micelle– With/without catalyst, slow/fast reaction depending on pH	∼40	Zhang et al. (2015)
Photo-induced crosslinking	– Technique used to stabilize polymers with functional groups that can polymerize– Reacting groups: coumarin or alkene– UV irradiation, photo initiator– Highly efficient, cytotoxicity concern	80–250	Zhang et al. (2015)

*Disulfide bonds* are found in natural peptides and proteins, assuring the rigidity and structural stability under certain conditions. Also, disulfide can reversibly reduce to thiol, as a function of thiol concentration of the environmental. Thiol-disulfide exchange is the principal reaction by which disulfide bonds are formed and rearranged in a protein and is a way for the preparation of recyclable cross-linking of micelles (Kakizawa et al., 1999).

For example, by using thiol-disulfide exchange, the dextran-lipoic acid assembly with encapsulated doxorubicin, is crosslinked with dithiothreitol in catalytic ratio, that reduces pyridyl disulfide (PDS) groups to thiols which further exchange with remaining PDS groups, while nanogels are obtained (Li et al., 2009).

Ryu et al. (2010) evaluated the potential to incorporate a hydrophobic guest molecule in biocompatible nanogels using intra/intermolecular disulfide bond formation of PDS containing polymers. The study indicated a rapid intracellular drug release over a short time due to the containing disulfide linkage that can be selectively biodegraded inside the target cell under the acidic conditions found in the endo/lysosomes and by cytoplasmic glutathione (Raemdonck et al., 2009). Compared to the conventional biodegradable delivery systems that allow a gradual release of therapeutic agents, these kinds of nanogels can render an enhanced therapeutic effect. Nanogels with disulfide bonds were also used to physically encapsulate siRNA during synthesis. Following cellular internalization, the nanogels disulfide bonds are cleaved, releasing siRNA.

The principal issue as respects the entrapment of biomolecules inside nanogels is represented by the possibility to compromise the bioactivity of the compounds during the loading process. In the case of biological macromolecules like growth factors, proteins or nucleic acid, it is preferable to adopt the method of physical loading by incorporating the biomolecules while synthesizing the nanogels. Drug loading and entrapment efficiency of therapeutic agents depend on the solubility in the polymeric matrix, while the drug release can be tuned by the particle size, the molecular weight and the copolymer composition of nanogels. It has been demonstrated not only that smaller particles with larger surface-to-volume ratio lead to rapid drug release, but also that by utilizing a polymer with higher molecular weight a slower *in vitro* release of drugs can be obtained (Gonçalves et al., 2010).

*Amine group* is usually used in the preparation of nanogels because of the reactivity toward carboxylic acids, activated esters, isocyanates, iodides and others. This technique provides an opportunity to introduce various stimuli-response properties into the nanogels by modulating the structure of the diamine crosslinker.

The research group of Wooley (Huang et al., 1998; Joralemon et al., 2005; Li et al., 2008) used a method to prepare shell-crosslinked knedel-like structures with amine crosslinkers. An amphiphilic block copolymer with crosslinkable hydrophilic poly(acrylic acid) block was synthesized. The assembly is crosslinked by amidation of carboxylic acid with diamine crosslinkers and the self assembly of the block copolymers.

*Imine bond* is one of the dynamic covalent bonds used to provide polymers with the abilities to adapt their structures or compositions in response to external stimuli (Lehn, 2007).

The imine bond appears as a result of the Schiff-base reaction occurring between aldehydes and amines or hydrazide containing compounds. It is used to generate biocompatible gels due to its mild reaction conditions. Also, the formation of imine bonds, stable under physiological conditions and labile at acidic pH, makes these nanogels promising candidates for the intracellular delivery of proteins (Zhang et al., 2015).

Fulton’ team used the imine bonds in the crosslinking of linear polymer chains into nanogel particles (Jackson et al., 2011). They also reported the preparation of nanogels with imine and disulfide bonds; the “demount” into the component polymer chains is triggered by the simultaneous application of two different stimuli (Jackson & Fulton, 2012). They used acrylamide-based linear copolymers displaying PDS attachments and either aldehyde or amine functional groups for nanogel preparation.

*Click chemistry* based cross-linking earned attention because of specificity, quantitative yield, tolerance to a broad variety of functional groups, and applicability under mild reaction conditions (Kolb et al., 2001; Tsarevsky et al., 2005).

The nanogel fabrication method reported by Wooley’ team (Joralemon et al., 2005; O’Reilly et al., 2005) is an example where click chemistry is applied. Starting from alkynyl shell functionalized block copolymer micelles based on the diblock copolymer poly(acrylic acid)-b-poly(styrene) as click-readied nanoscaffolds and azido dendrimers, the nanogel network is formed.

The polyion complex micelles with crosslinked core and thermoresponsive corona with stability against pH and salt, by click chemistry were also prepared (Zhang et al., 2008).

*Photo-induced* cross-linking is a “green” method as compared to chemical methods requiring cross-linking agents and/or catalysts and purified to remove the unreacted crosslinking agents and the residues. This method is used to stabilize polymer assemblies that are functionalized with polymerizable or dimerizable groups (Pioge et al., 2011). Although the photo-induced crosslinking is highly efficient, the initiator may induce cytotoxicity in the produced gels (Zhang et al., 2015).

In conclusion, the formation of nanogels by covalent crosslinking reactions induces the stability in a complex and unkind environment (e.g. *in vivo*), preventing the leakage of the load encapsulated drug and enhancing the therapeutic efficacy. On the other hand, traditional covalent crosslinking usually involves crosslinking agents, which may cause unwanted toxic effects and damage the entrapped delicate substances, such as cells, proteins, etc. Therefore, more and more biocompatible reactions are necessary in the biomedical field.

### Nanogel preparation by physical cross-linking

The physical self-assembly method supposes the controlled aggregation of a hydrophilic polymer capable of hydrophobic or electrostatic interactions, van der Waals forces of attraction and/or hydrogen bonding. The reaction takes place in mild conditions and in aqueous medium (Sasaki & Akiyoshi, 2010; De Robertis et al., 2015).

The stability of these nanogels is relatively less compared with that of the chemical cross-linked nanogels. Physically cross-linked nanogels are, for example, polysaccharides such as dextran, mannan, pullulan and polyaminoacids modified with cholesterol, derivative of chitosan with deoxycholic acid, etc. (Oh et al., 2008). In Table 3 are summarized some types of physical nanogels.

**Table 3.** Types of physical crosslinked nanogels.

Nanogels	Examples	References
Liposome modified	– Liposomes bearing succinylated polyglycidol undergo chain shrinking below pH 5.5 and deliver calcein to the cytoplasm.– Liposomes modified with poly(N-isopropylacrylamide) create temperature and pH sensitive nanogels, investigated for transdermal drug delivery.	(Kono et al., 1997; Roux et al., 2002)
Micellar	– Obtained by supramolecular self-assembly of amphiphilic block or graft copolymers in aqueous solutions.– Core–shell morphological structures obtained through hydrogen bonds, with core hydrophobic block segment surrounded by shell hydrophilic polymer block, that stabilizes the entire micelle.– Micelles’ core provides enough space for drugs/biomacromolecules encapsulation. The drug molecules in the hydrophobic core are protected from hydrolysis and enzymatic degradation.– N-isopropylacrylamide based micelle systems, evaluated as drug delivery devices.	(Rosler et al., 2001)
Hybrid	– Composite of nanogel particles dispersed in organic or inorganic matrices.– Ability to form complexes with various proteins, drugs and DNA; may coat the surface of liposomes, particles and solid surfaces including cells.– Able to deliver insulin and anticancer drugs.– Cholesterol-bearing pullulan composed of pullulan backbone and cholesterol branches. The molecules self aggregate and form stable nanogels through physical crosslinking points by the association of hydrophobic groups.– Nanogel in aqueous medium by self-assembly or aggregation of pullulan–poly(N-isopropylacrylamide), hydrophobized polysaccharides and hydrophobized pullulan.	(Akiyoshi et al., 2000; Kuroda et al., 2002; Lee & Akiyoshi, 2004)

## Trends in the nanogels’ bioconjugation

As it was mentioned before the nanogels meet about all the basic requirements of an adaptable nanocarrier delivery vehicle, when compared to other delivery systems (Vinogradov, 2010).

Due to the physicochemical structure, nanogels have: – deformability to enhance binding and retention within the targeting tissue, – improved stability due to the crosslinked structure to prolong their circulation time in the bloodstream, – a core–shell structure with a hydrophilic interior network, which allows both small-molecule or biomacromolecule drug loading and protection of hydrophilic compounds, – modular drug loading and release profiles, which can significantly enhance drug loading efficiency and bioavailability, and thus, reduce drug toxicity and side effects (Gonçalves et al., 2010; Eckmann et al., 2014).

The control of the drug release is obtained *via* conjugation of the nanogel surface with affinity ligands, antibodies or other molecules having molecular recognition specificity to the surface of nanocarrier, to target specific tissues expressing particular disease markers.

Taking into account the structural complexity of the biological systems, it is evident that the functional accessible diversity of synthetic polymer systems is limited. Each of these both classes – biological and synthetic polymers – has their own special characteristics and limitations (Cui & Gao, 2003).

**Figure 4.**
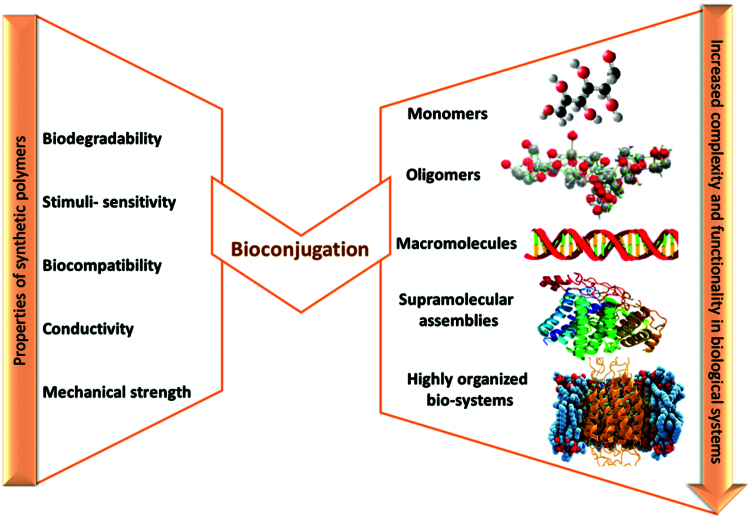
Complementary effect of synthetic and biological polymers in bioconjugation (adapted from Lutz & Börner, 2008).

The conjugation of biological macromolecules, such as: peptides, proteins, polysaccharides, oligonucleotides, enzymes, with synthetic polymers is an attractive and necessary topic in pharmaceutical chemistry to synthesize hybrid systems with synergistic properties, beyond both constituents, referred to as “polymer bioconjugates” (Figure 4) (Lutz & Börner, 2008; Börner 2009). Their attribute is to enhance the chemical properties, functions and to extend the range of applications in nanobiotechnology, drug delivery, gene therapy, tissue engineering, biosensors, etc.

These bioconjugate systems with complex hierarchically organized structure and controlled design at molecular level, must have uniform size and composition, and also water affinity for the use in biomedical domain. Their preparation with well defined self-assembled structures involves the use of the recent advances in polymer chemistry, a broad range of orthogonal methods, including controlled/“living” polymerization techniques as well as conjugation/activation chemistry (Eckmann et al., 2014; Vamvakaki, 2014). The synthetic approaches describing the synthesis of well-defined polymer bioconjugates are the topic of a great number of publications (Lutz & Börner, 2008; Börner, 2009; Kabanov & Vinogradov, 2009). With the emphasis on synthesis, properties and applications, these papers enable us to understand the connection between chemistry and the biological application of bioconjugated materials. Consequently this manuscript section is not extensive; some examples are highlighted and briefly discussed.

Nanogels are widely used in biomedicine as carriers for therapeutic agents. They have a remarkable swelling capacity in water environment and this determines an enhanced ability for drug loading, as compared with other nanocarriers, such as polymeric micelles, liposomes or nanoparticles. The nanogels offer a great loading space to incorporate not only small molecules of drugs, but also biomacromolecules. This higher loading skill can be obtained by self-assembly through a combination between electrostatic and hydrophobic interactions, in mild conditions, which is very important for the preservation of the biological activity of drugs and other biomacromolecules (proteins, polypeptides, etc.) (Vinogradov, 2007). Nanogels encapsulate proteins and thus improve their activity and stability (Yan et al., 2007; Ge et al., 2009). Also, the proteins encapsulated in nanogels show superior temperature and organic solvent stability. These important characteristics expand the catalytic and therapeutic potential of the bioconjugated nanogels. The binding of bioactive substances induces the collapse of the nanogel, which is reflected by the decrease in size. But the drug-nanogel particles remain dispersed due to functional groups exposed on the surface which form a protective hydrophilic layer around the nanogel particles, thus preventing the phase-separation.

The main methods by which the bioactive agents can be incorporated in nanogels are: covalent conjugation, physical entrapment and controlled self-assembly.

*Covalent conjugation* of biological agents is accomplished by using preformed nanogels or during nanogel synthesis. For example, Khmelnitsky et al. (1992) have covalently immobilized enzymes modified with acrylic groups in polymeric nanogels based on acrylamide and N,N-methylene bisacrylamide copolymers prepared in inverse microemulsion.

In other study, the chemotherapy agent cisplatin is covalently linked at the carboxyl groups in the nanogel made of crosslinked poly(ethylene glycol)-b-poly(methacrylic acid) (Oberoi et al., 2012). Biodistribution, antitumor efficacy and toxicity are evaluated in a mouse model. Cisplatin-loaded nanogels exhibit prolonged blood circulation, increased tumor accumulation and reduced renal exposure.

Matsumoto et al. (2013) studied the covalent conjugation of bovine serum albumin (BSA) to disulfide cross-linked polymeric nanogels based on poly(ethylene glycol) methyl ether methacrylate and PDS methacrylate with dithiothreitol. They established a nanogel system that can be used as an effective platform for the development of complex drug delivery systems. These compounds can encapsulate small lipophilic molecules on the interior and conjugate proteins by covalent attachment on the exterior of the nanogel.

*The physical entrapment* of drugs is by far the most commonly used loading method for drug delivery applications. The best incorporation strategy for an efficient entrapment must be selected according to the physicochemical characteristics of the drug–carrier pair. Several methods have been used for drug loading, such as dialysis, nanoprecipitation, solvent displacement/evaporation, desolvation or direct dissolution. The encapsulation efficiency is different for each specific nanosystem; thus, the chemotherapy agent taxol is encapsulated in poly(lactide-co-glycolide) nanodevices (Mu & Feng, 2003) with 100% efficiency and in poly(ɛ-caprolactone) (Kim & Lee, 2001) nanodevices with 20% efficiency, respectively.

Akiyoshi et al. (1998) used the physical entrapment of protein drugs in a nanogel based on hydrophobized cholesterol-bearing pullulan (CHP) in water. Insulin is spontaneously and easily complexed with the hydrogel nanoparticles, while the thermal and enzymatic denaturation and subsequent aggregation are effectively concealed upon complexation, thus being protected from enzymatic degradation.

In another study, Lee et al. (2007) synthesized a nanogel based on HA that physically entrapped small interfering RNA (siRNA) during the emulsion/crosslinking process.

Also the hydrophobic molecules are incorporated into the domains formed by hydrophobic chains present in the nanogels. But, these cases do not show a degree of bioactive substance loading greater than 10%. Kato et al. (2007) studied a nanogel based on CHP entrapping prostaglandin E2, which induces bone formation and has potential applications in the bone medicine.

The nanogel based on the copolymer of N-isopropylacrylamide and N-vinylpyrrolidone cross-linked with N,N′-methylenebisacrylamide was used by Soni et al. (2006) as a carrier to encapsulate N-hexylcarbamoyl-5-fluorouracil and to target the brain tissue.

The nanogel particles prepared by inverse emulsion photopolymerization of acrylated poly(ethylene glycol)-bl-poly(propylene glycol)-bl-poly(ethylene glycol) and poly(ethylene glycol) were investigated. The hydrophobic poly(propylene glycol)-rich nanodomains are suitable for incorporation by physical entrapment of hydrophobic drugs, such as doxorubicin up to 9.8% (Missirlis et al., 2005).

*Controlled self-assembly* of polyelectrolyte-based nanogels with oppositely charged biomolecules can produce nanogels with high content of biological agents. Self-assembly is the process in which the components are independently organizing into structurally well-defined stable aggregates. Molecular self-assembly is realized by diffusion and specific association of molecules through non-covalent intermolecular interactions, including electrostatic interactions and/or hydrophobic associations. Taken separately these interactions are weak, but dominate the structural and conformational behavior of the assembly due to the large number of involved interactions (Zhang, 2002; Gonçalves et al., 2010).

Bronich et al. (2001) studied a nanocomposite material synthesized by reacting nanoscale networks of hydrophilic nonionic and cationic polymers, poly(ethylene oxide)-*cl*-polyethyleneimine, with anionic surfactant: sodium tetradecyl sulfate. The formation of hydrophobic domains from polyethyleneimine (PEI)–surfactant complexes leads to the network collapse revealed by the decrease of the particle size from ca. 300 nm to ca. 50 nm. Due to their crosslinked architecture, the poly(ethylene oxide)-*cl*-PEI-based complexes are resistant to the changes in the environmental characteristics, such as pH and salt concentration. Poorly soluble biologically active molecules, retinoic acid (RA) and indomethacin, are also immobilized in this network. The nanogels loaded with RA, for example, form stable aqueous dispersions at physiological pH, recommended in pharmaceutical formulation.

Moreover, the nanogel formulations can be chemically modified to incorporate various ligands for targeted drug delivery or triggered drug release. The drug release from nanogel-drug conjugates is considerably extended and can be controlled by ligand stability. Preclinical studies suggest that nanogels can be used to efficiently deliver biopharmaceuticals even in cells, as well as to increase drug delivery across cellular barriers.

## Bioconjugated nanogels as versatile nanocarriers

Over the years, various review articles have reported the rapid development of nanogels as carriers for biomedical applications (Soni et al., 2016). Nanogels have become attractive as versatile nanocarriers for encapsulation and delivery of bioactive compounds such as drugs (Tiwari et al., 2015) or biological macromolecules (Arnfast et al., 2014) with a capacity to respond to the external physical or chemical signals like temperature and pH assignable to their nano-scaled size (Maya et al., 2013). Moreover, due to their extremely reduced size, nanogels have enhanced permeability and retention (EPR) effect (Ding et al., 2011). This property is important when the entrapped bioactive molecules or therapeutic agents such as siRNA, enzymes, and peptides can be easily inactivated and need to be safely delivered into the cytoplasm of the target cell.

However, the main limitation of using nanogels as targeted delivery systems is represented by their low target site specificity (Eckmann et al., 2014). Therefore, by conjugation of nanogels or nanogel compounds with biomolecules such as ligands (antibodies, enzyme), proteins or other molecules having molecular recognition specificity, the specificity for targeted delivery will improve (Soni et al., 2016). The attachment of biomolecules can also allow a rapid internalization of nanogels into the cells through endocytosis (Shimoda et al., 2011). In this chapter, we especially aim to overview the recent progress of bioconjugated nanogels designed for medical applications, with a particular emphasis on delivery of more fragile bioactive molecules such as proteins, peptides, antibodies, growth factors, vaccines, antisense oligonucleotides or nucleic acids, which are unstable and easily inactivated.

### Nanogels for intracellular delivery of genetic material (siRNA, DNA, oligodeoxynucleotides)

Nowadays, gene therapy designed for delivery of antisense oligodeoxynucleotides (ODNs), plasmid DNA (pDNA), siRNAs and micro RNAs (miRNAs) used in targeted inhibition of specific mRNA sequences has emerged as one of the most promising method to treat and diagnose numerous diseases like cancer, neurodegenerative disorders and viral infections (Soni et al., 2016).

However, the major challenges in designing an intracellular gene delivery system reside in crossing the cell membranes without being premature degraded by endogenous enzymes and providing a controlled release of the genetic material into the cell nucleus without inducing cytotoxicity and an immune response following degradation (Soni et al., 2016). A variety of gene delivery systems are available to be used in therapeutic gene transfer to restore a specific gene function or to knock down the expression of special genes. There are different viral and nonviral vectors for gene delivery and each of the delivery systems has some advantages and disadvantages. Although the use of viral vectors such as retrovirus, adenovirus (types 2 and 5), adeno-associated virus, herpes virus, pox virus and lentivirus has many advantages (high transfection rate and a faster transcription of the foreign material enclosed into viral genome delivery) (Nayerossadat et al., 2012), the non-viral vectors can be easily produced in large proportions, have higher genetic material carrying capacity compared with viral system, are targetable and can be administered repeatedly with minimal host immune response (Jere et al., 2009). The non-viral vectors for gene delivery made of cationic carriers such as cationic polymers/copolymers, lipids, liposomes, peptides, surfactants have been widely used to efficiently deliver therapeutic genetic material within cells (Nayerossadat et al., 2012). In the last few years, nanogels have shown promising utility as non-viral carriers, application ensured by high extracellular stability, high transfection efficiency, low toxicity and low immunogenicity of nanogels (Costa et al., 2015; Karimi et al., 2016).

Nanogels can be further conjugated with specific targeting biomolecules which by mixing with nucleic acid/genetic materials enable formation of complexes as lipoplexes or polyplexes via electrostatic interactions, enhancing the transfection of nucleic acids into cells and their stability under physiological conditions (Sunasee et al., 2012). In this context, Li et al. investigated the potential use of ethylenediamine (ED)-functionalized low-molecular-weight PGMA nanogels (PGED-NGs) as effective siRNA and pDNA carrier. To enhance pDNA and siRNA transfection, a natural antioxidant, lipoic acid (LA) was introduced into ED-functionalized PGMA and crosslinked to produce cationic reducible PGED-NGs with plentiful disulfide linkages. These nanogels crosslinked with disulfide linkages compared with unmodified PGED showed better performance of pDNA transfection and target-specific intracellular delivery of MALAT1 siRNA (siR-M) into hepatoma cells, suppressing cancerous cell proliferation and migration (Li et al., 2016).

**Figure 5.**
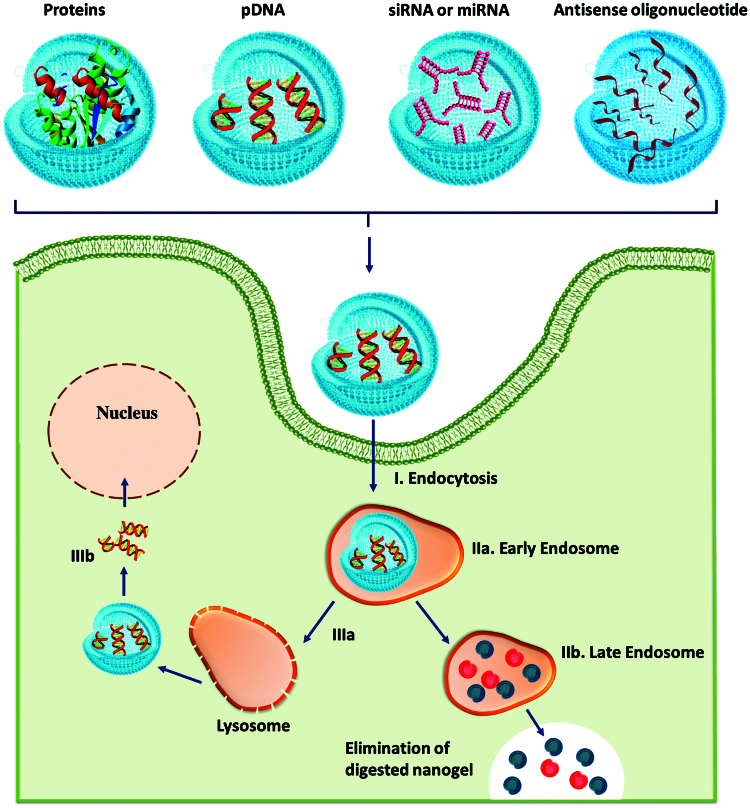
Intracellular delivery stages of biological macromolecules from nanogels (adapted from Keles et al., 2016).

General schemes of pDNA transfection and gene silencing mechanism of siRNA are illustrated in Figure 5. In all cases, after the cellular uptake and endosomal escape *via* the “proton sponge effect”, the release of biological macromolecules is mediated by the cytoplasmic enzymatic degradation of nanogels (see Figure 5) (Keles et al., 2016). Following nanogel degradation, pDNA and siRNA are released and take different routes. While pDNA (Figure 5a) is delivered into the cell nucleus, the siRNA (Figure 5c) is discharged into the cytosol; thus siRNA (Figure 5c) is recognized by the appropriate argonaute protein (AGO) within the RNA-induced silencing complex (RISC), which catalyzes the binding and cleavage of a specific messenger RNA (mRNA) sequence and inhibits the translation of proteins.

Hong et al. (2012) prepared siRNA/linear polyethyleneimine (LPEI) nanogels by electrostatic complexation and crosslinking between thiol-terminated siRNA and thiol-grafted LPEI. This new approach allows synthesizing nanogels with reductively cleavable linkages containing inter- and intra-molecular networks that will imprint stability to the nanogel network and will dissociate upon exposure to a reductive condition/environment (cytoplasm) releasing biologically active, free monomeric siRNA that can effectively initiate RNAi-mediated gene silencing for the sequence-specific cleavage of target mRNAs. The crosslinked siRNA/LPEI nanogel demonstrated significantly enhanced cellular uptake and gene silencing efficiency by comparison to the siRNA/LPEI complexes without crosslinks or with only LPEI-mediated crosslinks. Hence, siRNA/LPEI nanogels have the potential to be employed as an intracellular delivery system for siRNA therapeutics. Over the years, it has been demonstrated that branched PEI can be considered as an effective siRNA carrier because of its highly positive charges that allow it to form polyplexes with the negatively charged siRNA through strong electrostatic interaction (Soni et al., 2016).

However, the cytotoxicity of PEI, the increased diameter of the polyplex that can range from 180 to over 800 nm and the leakage of siRNA from PEI polyplex had limited the clinical use of PEI nanogels. Thus, these disadvantages have led to new approaches to tailor PEI-based nanogel properties while retaining their transfection efficiencies for gene delivery. Mimi et al. (2012) reported the preparation of PEI-based core–shell nanocarriers for siRNA delivery based on branched PEI layer as a shell conjugated to the preformed biodegradable gelatin nanoparticles.

The PEI/gelatin nanogels had spherical shape with an average diameter of 200 ± 40 nm, reduced toxicity (four-times) compared to the native PEI and were able to effectively deliver the siRNA into HeLa cell. Furthermore, the gelatin/PEI nanocarriers exhibited a considerable gene silencing effect through efficient delivery of siRNA, process that was considerably higher than that of the commercial available transfection agent, Lipofectamine^TM^ 2000 (32.0%±8.5%). The study results indicated that biocompatible and biodegradable core–shell nanogels containing PEI shells and compact gelatin cores can be employed as a new class of delivery carrier for siRNA therapeutic agents.

Similarly, Park et al. (2016) synthesized HA-shielded PEI/pDNA nanogels for receptor-mediated gene delivery and instead gelatin it utilized a HA core to conjugate branched PEI. Anionic pDNA was stabilized into the network by complexation with cationic PEI via electrostatic interactions. As a result, HA shielding of PEI/pDNA nanogels ensured an effectively internalization of nanogels into human mesenchymal stem cells (hMSCs) and HeLa cells via receptors located in the plasma membrane, recommending these nanocarriers as gene delivery vehicles to induce stem cell differentiation.

Dispenza et al. (2014) reported the formulation and characterization of biocompatible PVP-AA nanogels surface bioconjugated with a single strand oligonucleotide (ODN). The bioconjugated nanogels exhibited colloidal stability and an excellent ability/capacity to bypass cell membrane, accumulating in perinuclear area of the cytoplasm. Thus, it is reasonable to say that ODN-bioconjugated PVP-AA nanogels are promising nanocarriers for intracellular delivery of genetic material for a therapeutic effect.

### Nanogels for specific targeted protein delivery

Recent advances in medicine have highlighted the promising capacity of many proteins and peptides to be employed as therapeutic agents (Bruno et al., 2013). Despite this development, the major problems in using proteins and peptides as therapeutic agents still are the stabilization of proteins in delivery reservoirs at physiological pH values and temperatures and the proper design of protein carriers for the sustained and targeted delivery (Solaro et al., 2010; Muheem et al., 2016). One of the approaches in overcoming these limitations is to entrap proteins into hydrogel nanoparticles (nanogel), which can reduce denaturation of proteins by forming a colloidal stable complex with proteins at the nanometer scale (<50 nm) (Ayame et al., 2008).

Among the nanogels reported so far, the ones formed by self-assembly of hydrophobic group-modified water-soluble biopolymers such as cholesterol-hydrophobized pullulan or dextran have arisen as promising drug–carriers in protein therapies (Hasegawa et al., 2009).

In this context, Shimoda et al. (2011) explored the potential application as protein carrier of Arg-Gly-Asp (RGD)-modified cationic nanogels obtained by the self-assembly of ethylene diamine and cholesteryl group-modified pullulan (CHP). RGD is a cell recognition motif that allows a better efficacy of intracellular delivery. In this study, RGD-conjugated nanogels were efficiently uptaken by HeLa cells via integrin receptor-mediated endocytosis, specifically clathrin-mediated endocytosis and macropinocytosis. Nguyen et al. (2011) designed a disulfide-crosslinked nanogel by the self-assembly and oxidation of thiolated heparin-pluronic conjugate (DHP). Pluronic was conjugated to heparin, a negatively charged polysaccharide, to enhance the capacity for the encapsulation of biological drugs. Also, to avoid denaturation of protein in the blood stream and to increase the carrier stability, the nanogels were crosslinked with disulfide linkage. In this study, RNase A was used as a model protein to investigate efficacy of the protein delivery of DHP nanogels due to its specific or electrostatic interactions with heparin. The DHP nanogel had a reduced hydrodynamic size and high-drug loading efficiency. Also, the cytotoxicity assay indicated that DHP nanogels were more effective for the intracellular delivery of RNase A compared to non-crosslinked nanogel.

Additionally, Yang et al. (2014) reported the encapsulation of vascular endothelial growth factor (bFGF) and pDNA encoding VEGF165 genes within heparin-conjugated supramolecular pluronic nanogels pre-coated with PEI to study cells differentiation and proliferation. HPebFGF/PEI nanogels encapsulated with bFGF and VEGF165 pDNA gene complex were uptake by human endothelial progenitor cells (EPCs) most likely due to the conjugated heparin that facilitated cell membrane penetration. Thus, HPebFGF/PEI nanogels had then the capacity to efficiently promote endothelial cell differentiation and regeneration of vessels in the tissues of an ischemic limb model system.

Matsumoto et al. (2013) reported the conjugation of BSA to the surface of disulfide cross-linked polymeric nanogels encapsulated with lipophilic dye (DiI). They functionalized BSA with a thiol linker, and conjugated to the PDS moieties exposed at the nanogel surface. The results highlighted that this nanogel system is a simple and effective platform for targeted delivery of sophisticated therapeutic agents such as protein or antibodies into cytoplasm (see Figure 5b). Another biopolymer used in designing targeting carriers for medical application is HA. Besides being a biocompatible and biodegradable polymer, HA has been shown to promote angiogenesis in various types of tumors, and HA receptors such as CD44 and RHAMM are highly overexpressed in cancer cells (Park et al., 2012). Therefore, Weng et al. (2014) prepared HA conjugated with epigallocatechin-3-gallate (HA–EGCG) and used this modified biopolymer to prepare novel ternary nanogel via self-assembly for the targeted intracellular delivery of Granzyme B (GzmB) into cancer cells. EGCG is the main component of green tea catechins with anti-cancer properties; it has been demonstrated to bind physically to many proteins. Also, the GzmB protein was utilized for its capacity to mediate cancer cell apoptosis. The *in vitro* cytotoxicity assay indicated that GzmB-encapsulated nanogels had targeted toxicity against CD44-overexpressing HCT-116 cancer cells, while CD44-deficient cells showed little cytotoxic effect. The toxicity was attributed to GzmB-mediated apoptosis, indicating the potential use of HA–EGCG as effective intracellular protein carriers for targeted cancer therapy (Liang et al., 2016).

### Bioconjugated hydrogel nanoparticles as vaccine delivery or adjuvant systems

As it has been highlighted in the previous sections, the nanogel structure and properties can be readily tailored to encapsulate different kinds of molecules. In the last years, multi-responsive polymeric nanogels have become a promising new generation of vaccine delivery/adjuvant systems capable of triggering innate immune response or enhancing antigen delivery (Goncalves et al., 2016). Therefore, like in the case of genetic material and protein encapsulation, nanogels intrinsic properties allow protecting vaccine antigens from degradation *in vivo* and, by bioconjugation with antibodies or specific ligands, could increase active targeting specificity (Ferreira et al., 2013). Among them, polysaccharide-based nanogels such as cationic cholesterol-bearing pullulan (cCHP) appear to be very appealing as vaccine delivery systems due to their great biocompatibility and the abundance in unprocessed sources (Li et al., 2013).

Durán-Lobato et al. (2014) developed an oral vaccine delivery system based on poly(2-hydroxiethyl methacrylate-*co*-methacrylic acid) P(HEMA-*co*-MAA) nanogels functionalized with mannan. Nanogels bioconjugation on the surface with mannan was designed with the purpose to enhance M cell uptake and target C-type lectin receptors (CLRs) on antigen-presenting cells (APC) by mimicking carbohydrate moieties found on the surface of pathogens. The bioconjugated nanogels demonstrated not only pH-sensitive properties but also ensured an enhanced entrapping and protecting of the loaded material at low pH values, and initiated protein release after switching to intestinal pH values. Surface functionalization with mannan led to an enhanced uptake by macrophages as well as increasing the expression of relevant costimulatory molecules. These results indicate that the surface conjugation of P(HEMA-*co*-MAA) nanogels with mannan as carbohydrate moieties to provide “pathogen-like” features is a promising approach to prepare a more efficacious oral vaccine system.

Vitamin A, an essential micronutrient, has long been known to influence innate and adaptive immunity. Vitamin A or its active metabolite, RA, has been shown to control gene expression in a variety of processes including immune function by enhancing antigen-specific antibody production, CD8^+^ effector T cell activation and mucosal immunity (Raverdeau Mills, 2014). The immunoregulatory effect of vitamin A is mainly mediated by dentritic cells (DCs), in which vitamin A (retinol) can be converted by retinal dehydrogenases to its principal biologically active metabolite, all-trans retinoic acid (ATRA); ATRA regulates cell differentiation and its biological function is ensured through binding to its nuclear RA receptors expressed in lymphoid cells (Cassani et al., 2012). Wang et al. (2016) developed and evaluated pH-sensitive galactosyl dextran-retinal (GDR) nanogels as self-adjuvanted vaccine delivery system; dextran was bioconjugated through a pH-sensitive hydrazone bond with ATRA. Following bioconjugation, the nanogels were galactosylated to acquire dendritic cell (DC)-targeting ability. The GDR nanogels not only promoted DC maturation and antigen uptake, but also induced lysosomal rupture in DCs facilitating cytosolic antigen release. Accordingly to the results, GDR nanogel behaved as a self-adjuvanted nanocarrier greatly improving vaccine-induced anti-cancer immune responses. Toll-like receptors agonists (TLRs) are a family of surface molecules that function as primary activators of the innate immune system and are promising as vaccine adjuvant and for anticancer immunotherapy (Bohannon et al., 2013; Vasilakos & Tomai, 2013; Maisonneuve et al., 2014).

The main issue in using TLRs in soluble form is represented by the rapid entering in the systemic circulation that will lead to systemic inflammatory toxicity. Nuhn et al. designed a vaccine nano-carrier by conjugating a small molecule like imidazoquinoline-based TLR7/8 agonist to 50-nm-sized degradable polymeric nanogels prepared by self-assembly of amphiphilic block copolymers. The amphiphilic block copolymers were composed of a hydrophilic part, poly(ethylene glycol) (PEG)-like polymer block based on methoxy triethylene glycol methacrylate (mTEGMA) and a hydrophobic polymer block based on pentafluorophenyl methacrylate (PFPMA). Immunization studies on mice have highlighted that imidazoquinoline-bioconjugated nanogels were more potent at inducing T-cell responses and antibody responses against tuberculosis antigen when compared with soluble IMDQ. This approach demonstrates the potential of these IMDQ nanogels to be used as adjuvants for vaccination (Nuhn et al., 2016).

## Conclusions and future perspective

We have discussed here various facets regarding nanogels and their applicability in the biomedical domain. Undoubtedly, in the last years it has been demonstrated that nanotechnology applied to medicine can provide better ways of investigation and diagnostics with therapeutics of different diseases. Thus, it can be said that this area of research has developed spectacularly from day to day. This paper has reviewed the important aspects regarding nanogels with biomedical applications: the type of network crosslinking, the main characteristics of nanogel structures that make them appropriate to a variety of applications (swelling capacity, large surface area, stimuli sensitivity, the ability to efficiently encapsulate therapeutics and release them upon an environmental stimulus), bioconjugation and encapsulation of bioactive substances and methods of preparation, too. A special section is dedicated to nanogel carriers with promising applications in the leading areas of the biomedical research, such as: intracellular delivery of genetic material, specific targeted protein delivery and vaccine delivery.

It is obvious that researchers focus primarily on finding new nanogel structures with more and more capabilities: improved design to upload/release bioactive substances over a specified period of time and targeting properties to enable highly selective uptake into the desired organs.

While nanogel concept registered a noteworthy evolution, an urgent necessitate for relevant clinical data and a substantial number of unsolved issues regarding their pharmacodynamics, metabolism and pharmacokinetics, still need to be overcome, before nanogels can completely make the transition from clinical trial to current clinical application. At that point in the near future, nanogels as bioactives’ delivery carriers would improve the efficiency of medical care and benefit of the patients.
